# The causal effect of educational attainment on stress urinary incontinence: a two-sample mendelian randomization study

**DOI:** 10.1186/s12905-023-02724-2

**Published:** 2023-11-02

**Authors:** Shufei Zhang, Mao Chen, Jianfeng Liu, Lian Yang, Hanyue Li, Li Hong

**Affiliations:** Department of Obstetrics and Gynecology, Renmin Hospital of Wuhan University, Wuhan, Hubei Province 430060 People’s Republic of China

**Keywords:** Stress urinary incontinence, Mendelian randomization, Educational attainment, Years of schooling

## Abstract

**Background:**

Stress urinary incontinence (SUI) is characterized by involuntary urine leakage in response to increased abdominal pressure, such as coughing, laughing, or sneezing. It significantly affects women’s quality of life and imposes a substantial disease burden. While pregnancy and childbirth have been previously identified as risk factors for SUI, educational attainment may also play a role. Therefore, this paper investigates the causal relationship between educational attainment and SUI using two-sample Mendelian randomization (TSMR) analysis, years of schooling (YOS), and college or university degree (CUD) as proxies.

**Methods:**

Summary statistics of YOS, CUD, and SUI were obtained from genome-wide association studies (GWAS), and TSMR analysis was applied to explore potential causal relationships between them. Causal effects were mainly estimated using the standard inverse variance weighting (IVW) method, and complementary and sensitivity analyses were also performed using multiple methods.

**Results:**

The results indicate that both YOS (OR = 0.994, 95% CI: 0.992–0.996; *P* = 7.764E-10) and CUD (OR = 0.987, 95% CI: 0.983–0.991; *P* = 1.217E-09) may have a negative causal effect on SUI.

**Conclusions:**

Improving educational attainment may go some way towards reducing the risk of SUI. Therefore, it is important to increase efforts to improve the imbalance in educational development and safeguard women’s health.

**Supplementary Information:**

The online version contains supplementary material available at 10.1186/s12905-023-02724-2.

## Introduction

Stress urinary incontinence (SUI) is a prevalent pelvic floor dysfunction in women that is characterized by the involuntary leakage of urine during activities that increase abdominal pressure, such as coughing, laughing, and sneezing [1]. In addition, epidemiological surveys show that the overall prevalence of SUI in women is as high as 46%, which has caused a heavy disease burden, and severe patients have to undergo surgery, which further aggravates the pain and economic burden of patients; therefore, it has become imperative to investigate its pathogenesis and risk factors, and to seek good treatment and prevention [2–4].

Various risk factors have been identified for the development of SUI, including pregnancy, childbirth, and sociobehavioral factors such as smoking and obesity [5, 6]. Furthermore, several studies have demonstrated a correlation between educational attainment and chronic diseases such as type 2 diabetes, as well as influencing factors associated with SUI, such as obesity and high BMI. However, no research has investigated whether educational attainment has a causal relationship with SUI [7, 8].

Mendelian randomization, an emerging method used to examine causality, has gained considerable attention in recent years. This approach offers advantages such as time and resource efficiency compared to randomized controlled trials, while minimizing the influence of confounding factors and reverse causality [9, 10]. In this study, we utilized YOS and CUD as proxies for educational attainment in the population. Through two-sample Mendelian randomization (TSMR) analysis, we aim to investigate the causal relationship between educational attainment and SUI. Our findings provide novel insights into the pathogenesis, prevention, and treatment of SUI.

## Materials and methods

### Study design

**Fig. 1:**
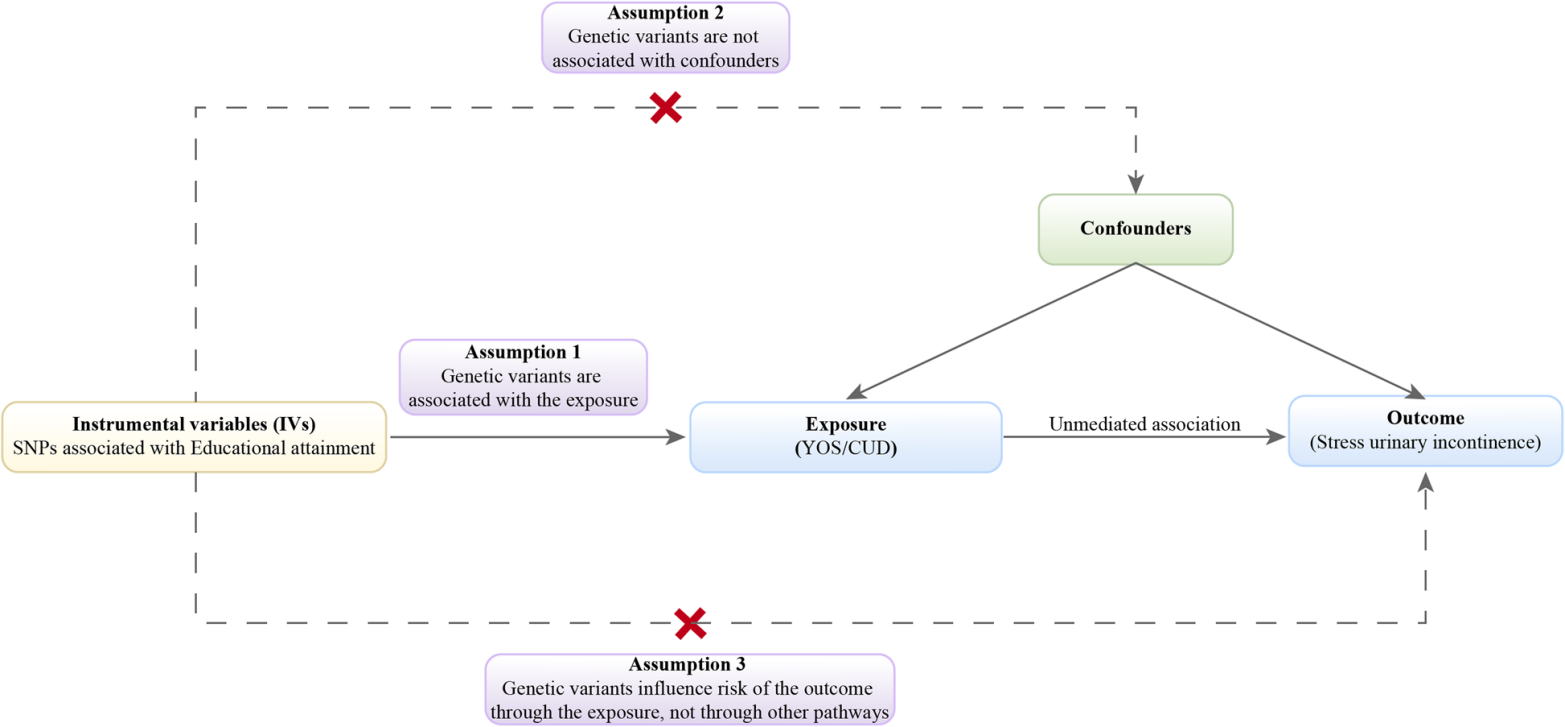
Schematic representation of the TSMR, which is based on three key assumptions: first, there is a significant association between genetic variance and exposure; second, there is no correlation between the instrumental variable (IV) and any confounding factors; and third, IV is only associated with SUI (outcome) through educational attainment (exposure) and not through direct association

This study was performed by TSMR with Single nucleotide polymorphisms (SNPs) obtained from the genome-wide association study (GWAS) pooled data (Fig. 1). Educational attainment was selected as the exposure, including YOS and CUD, while SUI was selected as the outcome. Mendelian randomization (MR) was performed with three key assumptions: (1) there is a significant association between genetic variation and exposure; (2) there is no correlation between instrumental variables (IV) and any confounding factors; and (3) exposure is the only way in which genetic variation affects the outcome of interest [11].

### Data sources

Two educational exposure factors were included in this study (Table 1). The IV for YOS was obtained from Lee’s GWAS, which included 766,345 individuals and identified 10,101,242 SNPs; the IV for CUD was obtained from Neale et al.‘s GWAS, which involved 334,070 individuals and identified a total of 10,894,596 SNPs [12]. Data related to SUI were obtained from a GWAS by Ben Elsworth et al. including 4340 European female cases and 458,670 European pedigree controls, and 9,851,867 SNPs were identified. Diagnosis of SUI based on main ICD10: N39.3. All data are available at https://gwas.mrcieu.ac.uk/ and this article does not contain any identifiable patient information and therefore does not require ethical approval.

**Table 1:** The characteristics of GWAS on the exposures and outcomes

Exposure	Consortium	Total population	Cases/controls	Ethnicity
Years of schooling	SSGAC	766,345	NA	European
Qualifications: College or University degree	Neale Lab	334,070	106,305/227,765	European
Outcome	Consortium	Total population	Cases/controls	Ethnicity
Stress urinary incontinence	MRC-IEU	463,010	4,340/ 458,670	European

### IVs extraction

A threshold of *p* < 5 × 10 − 8 was chosen to extract IVs, and to avoid bias due to linkage disequilibrium (LD), r^2^ = 0.001 and the number of bases between two SNPs (kb > 10,000) was set [13].

### Statistical analysis

The causal effect of education on SUI was estimated mainly by the standard inverse variance weighted (IVW) method, supplemented by MR-Egger, weighted median, simple mode and weight mode methods as well analysis [14]. Cochran’s Q test was subsequently performed to assess heterogeneity, with *P* > 0.05 assuming that there was no heterogeneity in the included IVs, ignoring the effect of heterogeneity on the estimation of causal effects, and if there was significant heterogeneity, the random effects IVW method was used (*P* < 0.05) [15, 16]. Bias due to horizontal pleiotropy was assessed by MR-Egger intercept test, i.e., MR-Egger regression analysis, with *P* > 0.05 to consider the possibility of weak genetic pleiotropy and ignore its effect [17]. Finally, the reliability of TSMR analysis results was assessed by the leave-one-out test [18]. All data analyses were performed in the TwoSampleMR package (R version: 4.2.1) [19, 20]. Differences were considered statistically significant when *P* < 0.05.

## Results

### Causal effect of YOS on SUI

**Fig. 2:**
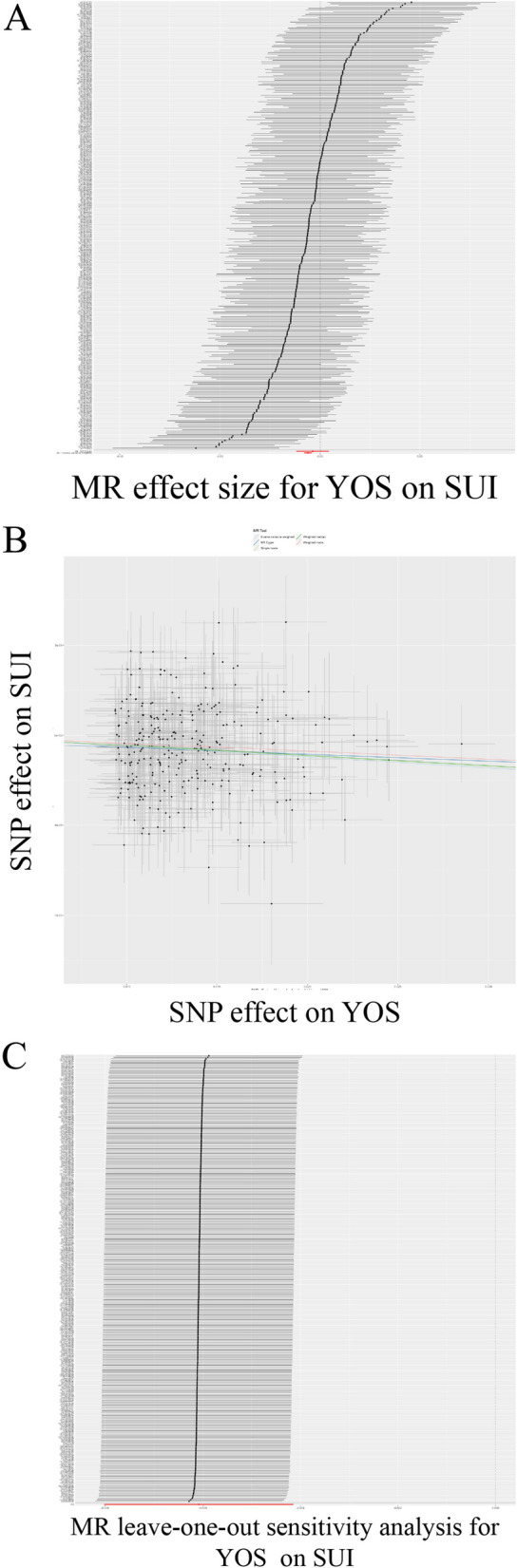
TSMR analysis of “Years of schooling” on SUI. **A** Forest plot; **B** Point plot; **C** Leave-one-out test plot

We first assessed the causal effect of YOS on SUI (Table 2; Fig. 2). The results of the IVW assessment showed a negative causal effect of YOS on SUI, with the risk of SUI decreasing with a 4.2-year increase in YOS (OR = 0.994, 95%CI: 0.992–0.996; *P* = 7.764E-10), WM (OR = 0.994, 95%CI: 0.992–0.997; *p* = 7.666E-05) also verified this result. Cochran’s Q test (Cochran’s Q = 301.259; *p* = 0.342) and MR-Egger regression (Egger intercept =-3.199E-05; *p* = 0.565) showed no heterogeneity or horizontal polymorphism, and the leave-one-out test showed reliable and stable results. The IVs involved are shown in SUP Table 1.

**Table 2:** Two-sample MR estimates of relationship between educational attainment and stress urinary incontinence

Exposure	MR Method	Stress urinary incontinence			Heterogeneity		Horizontal pleiotropy	
		No. of SNPs	OR (95% CI)	*P*-Value	Cochran’s Q	*P*-Value	Egger intercept	*P*-Value
Years of schooling	IVW	293	0.994 (0.992–0.996)	7.764E-10	301.259	0.342	-3.199E-05	0.565
	MR-Egger		0.996 (0.988–1.004)	0.363				
	WM		0.994 (0.992–0.997)	7.666E-05				
	Simple mode		0.994 (0.986–1.003)	0.186				
	Weighted mode		0.996 (0.988–1.003)	0.261				
Qualifications: College or University degree	IVW	171	0.987 (0.983–0.991)	1.217E-09	195.851	0.085	4.007E-05	0.659
	MR-Egger		0.982 (0.962–1.003)	0.102				
	WM		0.988 (0.982–0.994)	2.988E-05				
	Simple mode		0.984 (0.967–1.002)	0.087				
	Weighted mode		0.986 (0.971–1.001)	0.072				

### Causal effect of CUD on SUI

**Fig. 3:**
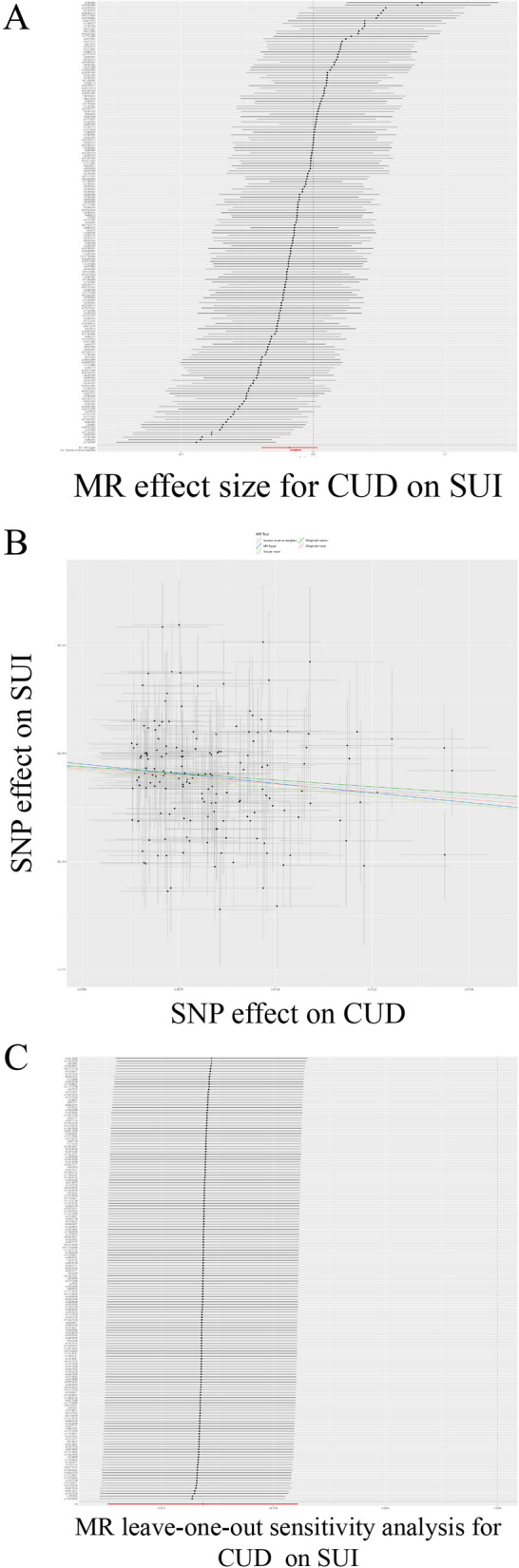
TSMR analysis of “Qualifications: College or University degree” on SUI. **A** Forest plot; **B** Point plot; **C** Leave-one-out test plot

Next, we assessed the causal effect of CUD on SUI (Table 2; Fig. 3). the results of the IVW assessment showed a negative causal effect of CUD on SUI, suggesting that the attainment of a higher degree may reduce the risk of developing SUI (OR = 0.987, 95% CI: 0.983–0.991; *P* = 1.217E-09), WM (OR = 0.988, 95% CI: 0.982–0.994; *p* = 2.988E-05) also validated this result. Cochran’s Q test (Cochran’s Q = 195.851; *p* = 0.085) and MR-Egger regression (Egger intercept = 4.007E-05; *p* = 0.659) showed no heterogeneity or horizontal polymorphism, and the leave-one-out test showed reliable and stable results. The IVs involved are shown in SUP Table 2.

## Discussion

The incidence of SUI remains high, and the burden of disease due to SUI is likely to increase further with the aging of the population, but the harm of SUI has been underestimated for a long time because it is a nonfatal disease [21]. In this study, we conducted a TSMR analysis using GWAS data to investigate the causal relationship between educational attainment (represented by YOS and CUD) and SUI. The results showed that hereditary increases in YOS were significantly associated with a reduced risk of developing SUI, with IVW showing a reduced risk of SUI for every 4.2-year increase in YOS (OR = 0.994, 95% CI: 0.992–0.996; *P* = 7.764E-10). YOS is largely representative of education level, but here again, we analysed separately causality of higher education on SUI and the results show a negative causality of CUD on SUI as well, which is consistent with the results presented above.

Examining the mechanisms underlying this negative causal relationship, one possibility is the influence of education on contraceptive use and early pregnancy. Limited access to education may contribute to factors such as lack of sex education, inadequate contraception, and early sexual activity [22]. Previous studies have demonstrated a higher prevalence of unintended pregnancies among adolescents with lower YOS, who are also less likely to use effective contraception [23]. Additionally, there is a significant negative genetic correlation between age at first childbirth and the number of children born [24]. Hence, increased educational attainment may promote contraceptive use, reduce the incidence of early pregnancy and multiple births, and subsequently mitigate pelvic floor damage, thereby lowering the risk of SUI.

While the risk of SUI increases with each childbirth, lower educational attainment may result in a lack of knowledge and awareness regarding the pelvic floor, reducing the likelihood of receiving pelvic floor education or rehabilitation [25]. Previous studies have shown that increased education positively impacts maternal health attitudes, and comprehensive postpartum care and rehabilitation can effectively address pelvic floor injuries associated with pregnancy and childbirth, preventing or treating SUI at an early stage, consistent with our findings [26, 27].

Moreover, educational level can significantly influence women’s fertility intentions and lifestyle choices. Fertility rates have undergone changes in various countries, and studies have reported a decline in fertility rates associated with increased female YOS [28]. College education plays a vital role in women’s careers, earnings, and health behaviors. A study from China revealed that higher levels of education reduce women’s willingness to have children [29, 30]. Another study showed that having a college or university degree reduces the likelihood of giving birth and the total number of births, possibly due to labor market-related factors negatively affecting fertility [31]. These findings align with our results, indicating that increasing educational attainment reduces fertility intentions and results in lower fertility rates. The decrease in total fertility may lead to a lower number of women experiencing birth injuries, consequently decreasing the risk of SUI. Additionally, individuals with longer YOS tend to adopt healthier lifestyles, including lower rates of smoking, alcohol consumption, and high BMI, further reducing the risk of SUI [32].

This study offers several strengths. First, it is the first study to specifically examine the causal relationship between educational attainment and SUI in women, representing an advancement in the field. Second, all the datasets utilized in our analysis were from individuals of European origin, potentially minimizing the impact of population stratification on the observed association. Finally, our study offers a multidimensional assessment of educational attainment, examining both the YOS and CUD levels. This approach provides a more comprehensive and in-depth analytical perspective, offering valuable evidence for reference in future research.

However, there are several limitations to our study. First, while our study yielded statistically significant results, the effect estimate is small. As a result, the causal effects derived from this study may not hold much practical significance. This phenomenon may be attributed to pleiotropy, and although we conducted pleiotropy tests and employed a random effects IVW model to address heterogeneity, it is currently not feasible to completely rule out pleiotropy in a study [33]. Additionally, due to the scarcity of data on SUI and educational attainment, we obtained GWAS data from the UK Biobank, resulting in some overlap in the study population. This may have introduced bias to some extent. More studies with diverse populations are needed in the future to further validate our findings. Furthermore, due to the aforementioned reasons, our data included both males and females, and we were not able to conduct a separate analysis specifically on female SUI patients. Future research should aim to include more data with gender information to support subsequent gender-stratified studies.

In conclusion, our study provides evidence suggesting a negative causal effect of educational attainment on SUI. However, it is important to note that even though an increase in educational attainment may lead to a decline in fertility rates, uneducated girls in certain economically deprived regions or countries may face a higher risk of early marriage, sexual violence, and child marriage [34]. This underscores the need for countries to address economic and educational disparities to safeguard women’s health.

## Conclusion

In this study, we employed TSMR analysis using YOS and CUD as proxy variables, and the results provide support for a potential negative causal relationship between educational attainment and SUI. This suggests that enhancing education levels can contribute to reducing the risk of developing SUI. Consequently, we should focus on improving the imbalance of educational development, increasing attention to female pelvic floor health problems, and maintaining female health.

### Supplementary Information

**Additional file 1: Supplementary Table 1.** IVs of "Years of schooling".

**Additional file 2: Supplementary Table 2.** IVs of "Qualifications: College or University degree".

## Data Availability

The reported data is also available with the corresponding author and can be accessed upon submitting a reasonable request.

## Abbreviations

SUI: Stress urinary incontinence
MR: Mendelian randomization
TSMR: Two-sample Mendelian randomization
YOS: Years of schooling
CUD: College or university degree
GWAS: Genome-wide association studies
IVW: Standard inverse variance weighting
LD: Linkage disequilibrium
SNPs: Single nucleotide polymorphisms
IV: Instrumental variables
